# Multiple risks for children of incarcerated mothers: A descriptive study

**DOI:** 10.23889/ijpds.v9i5.2594

**Published:** 2024-09-18

**Authors:** Megan Bell, Sharon Dawe, Leonie Segal, Matthew Spittal, Susan Dennison, Stuart Kinner, David Preen, Scott Sims

**Affiliations:** 1 University of Western Australia; 2 Griffith University; 3 University of South Australia; 4 University of Melbourne; 5 Griffith University; 6 Curtin University

## Objectives and Approach

Survey data suggests that children of incarcerated mothers are exposed to a range of risk factors that can have negative impacts on life-course outcomes. This retrospective cohort study uses a population sample to describe the extent and mix of risk factors to which children of incarcerated mothers are exposed. Administrative data from health, justice, child protective services, births, and deaths were merged for 9,380 children exposed to maternal incarceration and an unexposed comparison group of 22,716 children. Risk ratios and 95% confidence intervals were calculated for birth complications and adverse childhood experiences (ACEs) for children exposed to maternal incarceration compared to those not exposed, adjusted for sociodemographic risk factors. A multiple risk score summed the number of recorded risk factors (0-17) for children with and without maternal incarceration exposure.

## Results

Compared to unexposed children, children of incarcerated mothers had a higher risk of preterm birth, low birthweight, and all ACEs, including a 15-times (95%CI 14.2, 16.3) higher risk of exposure to maternal substance use disorders and 18-times (95%CI 16.8, 20.7) higher risk of placement in out-of-home care. Only 1% of children of incarcerated mothers did not experience any of the included risk factors, compared to 26% of unexposed children (p<0.001).

## Conclusions

Children of incarcerated mothers disproportionately experience multiple risks, especially maltreatment, placement in out-of-home care, and maternal substance use.

## Implications

There is a significant public health opportunity to identify and address the needs of children of incarcerated mothers at the points of maternal arrest, sentencing, and incarceration.

